# Prenatal sonographic diagnosis of urorectal septum malformation sequence and chromosomal microarray analysis: A case report and review of the literature

**DOI:** 10.1097/MD.0000000000005326

**Published:** 2016-11-11

**Authors:** Yan Pei, Qingqing Wu, Yan Liu, Lijuan Sun, Wenxue Zhi, Puqing Zhang

**Affiliations:** a Department of Ultrasound; b Department of Obstetrics; c Department of Pathology, Beijing Obstetrics and Gynecology Hospital, Capital Medical University, Beijing, China.

**Keywords:** chromosomal microarray analysis, fetal ultrasonography, prenatal diagnosis, *RBFOX1*, urorectal septum malformation sequence

## Abstract

**Introduction:**

Urorectal septum malformation sequence (URSMS) is a rare congenital abnormal syndrome that is caused by the incomplete division of the cloaca. Based on whether the cloaca membrane breaks down or not, the URSMS are classified as full and partial forms. The prenatal diagnosis of URSMS remains challenging because of poor recognition to this malformation and the relatively non-specific sonographic features. We report a prenatally sonographic diagnosed case of the partial URSMS, and review the literature to summarize the prenatal features.

**Case report and review:**

A 37-year old woman was referred at 24 weeks of gestation for fetal abdominal cyst. Detailed sonographic examination was done and revealed the vesicocolic fistula, distended colon, absence of perianal hypoechoic ring, pyelectasis, and small stomach bubble. The URSMS was suspected.

Amniocentesis was done and karyotyping revealed 46,XY. Furthermore, chromosomal microarray analysis (CMA) was performed for the first time in URSMS and an alteration of 111.8Kb deletion was detected in 16p13.3 which was located inside the RBFOX1 gene. Parental studies showed that the deletion was inherited from the father who has nomal clinical phenotype.

The woman elected to terminate the pregnancy at 25 weeks gestation and postmortem examination confirmed the diagnosis of partial URSMS.

The published studies were reviewed and 28 cases of URSMS with conducted prenatal ultrasonography were collected in this report. The most common sonographic description, as suspicious signs of URSMS, were severe oligohydramnios or anhydramnios, urinary tract anomalies, fetal intra-abdominal cysts, and dilated bowel. Also, enterolithiasis and vesicocolic fistula were relatively infrequent but highly specific feature of URSMS.

**Conclusions:**

URSMS is difficult to be diagnosed prenatally. However, it has characteristic features that can be detected by fetal ultrasonography, and a precise prenatal sonographic examination is crucial for diagnosing URSMS. Besides, more genomic profiling studies are needed to elucidate the causality.

## Abstract 2

Supplemental Digital Content is available in the text

## 1 Introduction

Urorectal septum malformation sequence (URSMS) describes a relatively rare congenital anomaly characterized by flat perineum with no perineal and anal openings, external genital defect, and urogenital and colonic abnormalities. It is assessed to occur in 1:50,000 to 250,000 births.^[1]^ The spectrum ranges from partial to full URSMS. Partial URSMS is a milder form with only 1 perineal opening as a common outlet and drain for feces and urine to the outside. The etiology of this condition remains unclear, but it has been proposed that the basic pathogenesis is the inadequate division of the cloaca and abnormal development of the urorectal septum.^[1]^ URSMS is difficult to be diagnosed prenatally. As far as we know, only a few cases of URSMS have been reported to date, and most cases are identified after delivery. Meanwhile, chromosomal microarray analysis (CMA) studies have not been conducted in this condition. In this study, URSMS was diagnosed prenatally and single-nucleotide polymorphism-based array (SNP-array) was performed for the first time to detect chromosomal aberration, and the sonographic signs of the 28 cases of URSMS from PubMed database were summarized.

## 2 Case report

**Figure 1:**
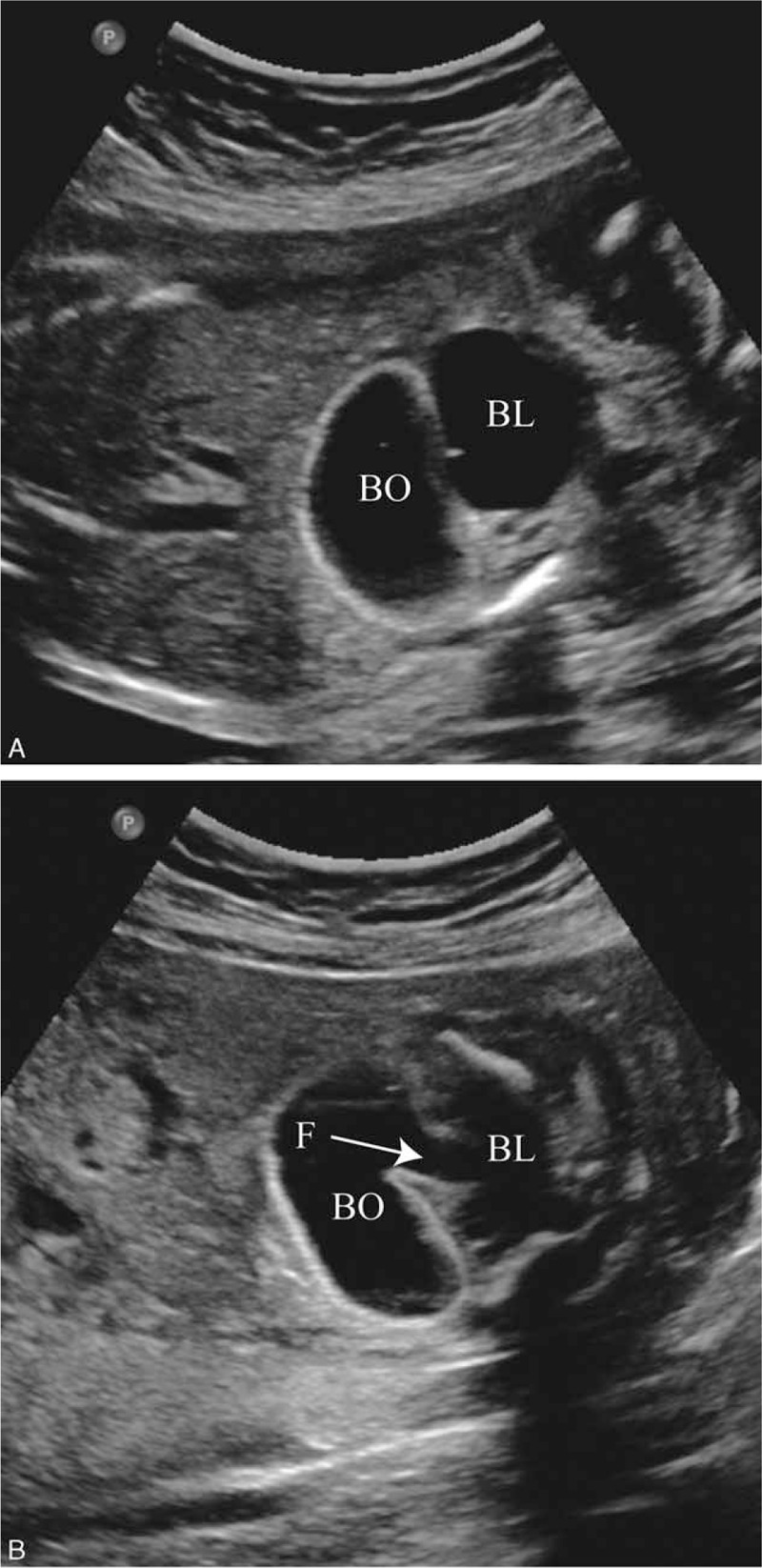
The ultrasound examination of urorectal septum malformation sequence. A, It shows distended bowel and bladder in the lower abdomen. B, The fistula is seen between bowel and bladder. BL = bladder, BO = bowel, F = fistula.

We report a case of URSMS in a fetus of a 37-year-old woman, primigravida, who was referred at 24 weeks of gestation for a further ultrasound scan. The previous sonographic results which were obtained from a district hospital at 16 weeks’ gestation showed a 2.3 × 1.0 cm left abdominal cystic mass and bilateral pyelectasis of the kidneys (left 5.2 mm, right 5.0 mm) with normal amniotic fluid. The history of the woman was unremarkable, and there was no family history of congenital anomalies. The mother denied teratogenic exposure. Serologic test for TORCH (Toxoplasmosis, Rubella, Cytomegalovirus, Herpes simplex virus) infection diseases was negative. Detailed sonographic examination had been conducted and revealed a fetus in transverse lie. The shape of the fetal skull was dolichocephaly, and nuchal fold thickness was 6.3 mm. There was a gradually dilated bowel and deflated bladder, and alternation between filling and emptying of the bowel and bladder was observed during the dynamic observation (see Video, Supplemental Video, which shows the process of the alternation between the bowel and bladder). The phenomenon raised the suspicion that there might be a fistulous connection between the bowel and the bladder. After about 30 minutes, the distended loop of colon (1.9 cm) was found connected to the bladder in the lower abdomen (Fig. 1). Also, the hypoechoic ring representing the anal sphincter was not visualized, which strongly indicates the anorectal atresia. The renal pelvis was separated (left 4 mm, right 6 mm). The amniotic fluid index was 4.1. Fetal stomach bubble remained considerably small with the diameter of 6 mm. A sonographic diagnosis of URSMS was made.

**Figure 2:**
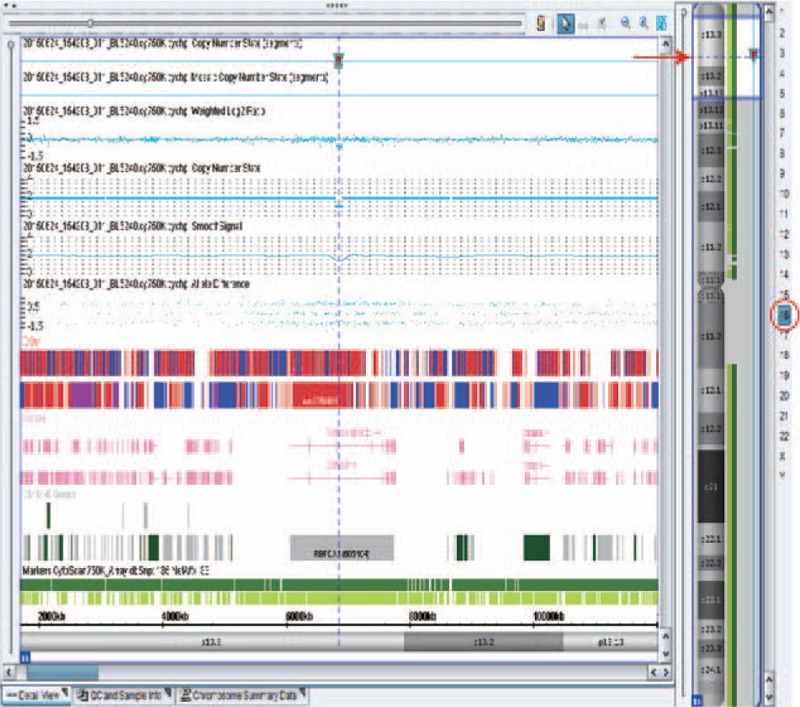
The SNP-array analysis results. A 118-kb deletion at chromosome 16p13.3, which is located inside *RBFOX1* gene. The red circle indicates chromosome 16, and the red arrow indicates cytoband of p13.3. The chromosome numbers and cytobands are shown and labeled on the right side. The view on the left side shows the detected segments, regions, and reference annotations in detail. The location of the chromosomal deletion segments is denoted by dotted-vertical line. SNP-array = single-nucleotide polymorphism-based array.

In consideration of the abnormal ultrasound findings, amniocentesis had been done and karyotyping revealed a normal male (46,XY with G-banding). Furthermore, CMA using SNP-array (Affymetrix CytoScan 750K Array) was performed, and a 111.8-kb deletion at chromosome 16p13.3 (6,798,232–6,910,094) was detected. This deleted segment was located inside the *RBFOX1* gene (OMIM ID: ∗605104) (Fig. 2). Parental SNP-array also had been done and the same deletion was detected in father who has normal clinical phenotype.

**Figure 3:**
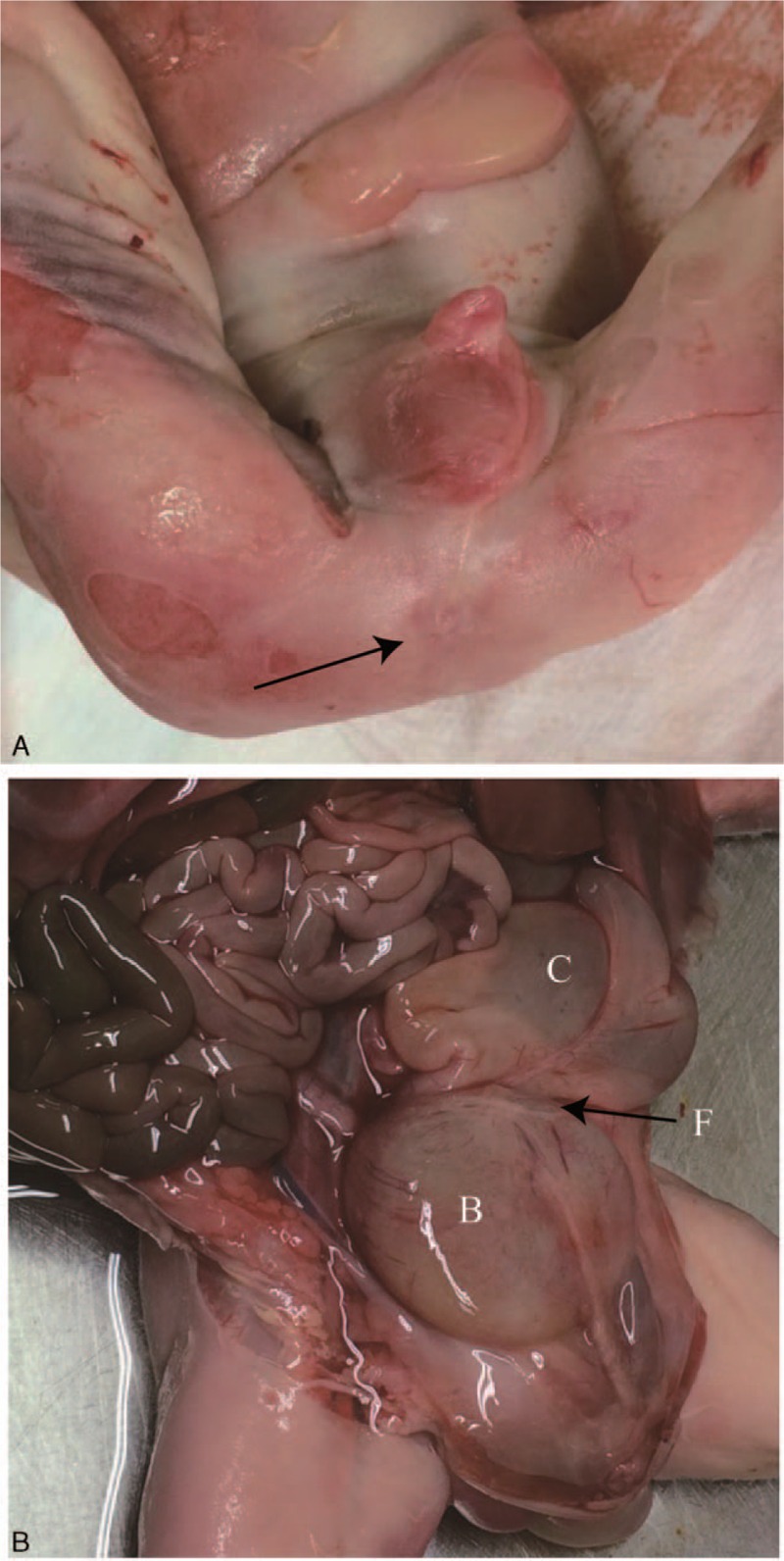
Gross inspection. A, It shows a single perineum opening at the tip of the penis that drained feces and urine, absence of anal orifice (arrow), and hypoplastic scrotum with median raphe absent. B, The distended colon opens to the urinary bladder by a fistulous connection (arrow). B = bladder, C = colon, F = fistula.

We informed the parents that the full URSMS was frequently lethal because the pulmonary hypoplasia was often caused by severe oligohydramnios, but the prognosis in the partial form was generally good in contrast to the full form. In consideration of the normal amniotic fluid index, the fetus was more likely to the partial form, and the prognosis would not be too poor. However, the woman still elected to terminate the pregnancy at 25 weeks’ gestation and the post mortem examination was performed. Gross inspections showed a single perineal opening at the tip of the penis that drained feces and urine with absence of anal orifice. Autopsy revealed that the scrotum was hypoplastic, with absence of median raphe. There was only 1 testis within the scrotum, and the other 1 was not found within the abdominal cavity or anywhere along the normal route of descent, such as the inguinal canal. Internally, the cecum was displaced into the left side. The colon was prominently dilated to 1.9 cm in diameter and opening directly to the dilated bladder to form a wide vesicocolic fistula (Fig. 3). The whole rectum was absent. The bladder was filled with the turbid fluid and contained meconium. The bladder was coalesced with the colon into a common channel of about 3 cm in length, which was connected to the external surface. The kidneys were normal, and the renal pelvis was dilated with the size of 0.8 cm left and 0.9 cm right. The widths of the posterior horn and the anterior horn of the lateral ventricles were 1.5 and 2.0 cm, respectively. The partial URSMS was diagnosed according to the findings of a single perineum opening, the common cloaca, vesico-colonic connection, anorectal atresia, distended bowel, and the single orifice. The post mortem examination findings were consistent with the prenatal sonographic findings. The study was approved by the Ethics Committees of Beijing Obstetrics and Gynecology Hospital, Capital Medical University. Apart from this, written informed consent was achieved from the patient.

## 3 Discussion

The URSMS is an extremely rare congenital malformation syndrome, which was first described by Escobar et al.^[1]^ It describes a constellation of abnormalities for the deficient separation of the cloaca and failure to rupture of the membrane. Malformations include lacking anal and perineal openings, external genital defect, and colonic and urogenital abnormalities. Wheeler and Weaver^[2]^ later proposed the milder type of URSMS named partial URSMS. In partial URSMS, the cloaca membrane had ruptured, whereas division of the cloaca was not completed. It is characterized by the presence of a single perineal outflow (any opening in the anterior or posterior perineum) that acts as an orifice for the common channel. There are different names for the URSMS which have been used in the literature, such as cloacal dysgenesis sequence,^[3]^ persistent cloaca,^[4]^ and cloacal malformation/anomaly. Recently, Tennant et al^[5]^ reported that the prevalence of partial URSMS was 2.8 per 100,000 total born, which increased considerably over time. The exact pathogenesis of the anomaly is unclear. Several experiments suggested the lower mesodermal defects and the abnormal development of the urorectal septum as the primary cause of the URSMS.^[2,6,7]^

Urorectal septum malformation sequence is difficult to be diagnosed prenatally. However, it has characteristic features that can be detected by fetal ultrasonography, such as enterolithiasis,^[8,9]^ distended bowel,^[10]^ fetal intra-abdominal cyst.^[11]^ Achiron et al^[10]^ reported that prenatal diagnosis of URSMS was feasible by using the ultrasonographic criteria of enlarged bowel with echogenic foci and ambiguous genitalia. Lubusky et al^[8]^ described the prenatal detection of enterolithiasis in the fetuses of URSMS and suggested it was a warning sign for bowel obstruction. To our knowledge, there were 15 reports (involved 28 cases of URSMS) describing prenatal ultrasound findings of URSMS (Table 1). Among these, 22 cases of them were full URSMS, and 6 were partial. In all, 28 cases of URSMS, the most common sonographic description, were severe oligohydramnios and oranhydramnios, which were observed in 22 patients. Most of them (21 of 22) were full URSMS, whereas only 1 case of the partial type occurred in this finding because a urethral opening existed in partial URSMS. Urinary tract abnormalities such as dysplastic kidneys, hydroureters, and hydronephrosis were also frequent features for obstruction of urinary opening, which were observed in 14 cases, and 13 of them were full URSMS. In addition, other frequent sonographic findings in the URSMS include megacystis/dilated bladder (9/28), fetal intra-abdominal cysts (8/28), dilated bowel (8/28), enterolithiasis (5/28), abdominal ascites (3/28), and fetal abdominal distension (3/28). In our reported case, sonographic findings of the abdominal cyst, vesicocolic fistula, and absence of perianal hypoechoic ring played key roles in prenatal diagnosis of URSMs.

**Table 1:**
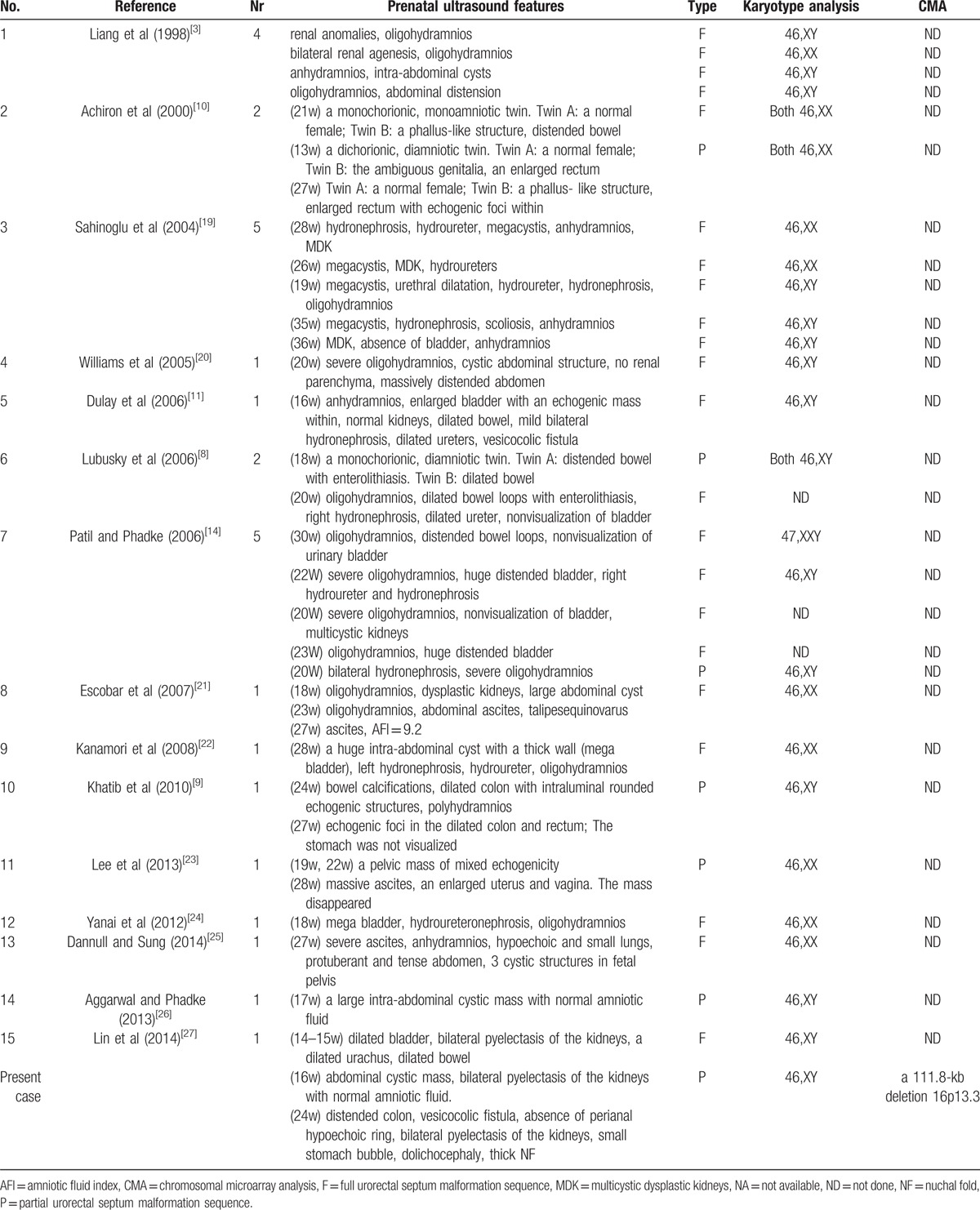
Summary of the prenatal ultrasound features associated with urorectal septum malformation sequence from literatures.

Chromosomal microarray analysis is used for identifying chromosomal abnormalities, including submicroscopic deletions and duplications [known as copy-number variants (CNVs)],which are too small to be detected by conventional karyotyping.^[12,13]^ Hence, the CMA is suggested to be a first-tier test in the evaluation of patients with neurocognitive disease and congenital abnormalities. However, CMA has not been conducted in the case of URSMS yet. Patil and Phadke^[14]^ reported karyotyping of 47,XXY in a case of URSMS, but considered it as coincidence. In our case, the karyotype was normal, but an alteration of 111.8-kb deletion was seen in chromosome 16p13.3, which was located inside the *RBFOX1*gene (OMIM ID: ∗605104). *RBFO*X1, also known as *A2BP1*, encodes a highly conserved RNA-binding protein that regulates tissue-specific splicing, indicating critical roles in development and differentiation.^[15]^ Loss of *RBFOX1* activity can cause malfunctions in the splicing of many genes, generating altered products that differ from those found in normal tissue. According to the DatabasE of genomiC varIation and Phenotype in Humans using Ensembl Resources (DECIPHER) database reports (https://decipher.sanger.ac.uk/) and literature, intragenic deletion of *RBFOX1*gene was found to be related neuropsychiatric and neurodevelopmental disorders,^[16]^ and also colorectal and lung tumorigenesis.^[15,17]^ However, through the parental studies, the CNVs detected in the fetal samples was found to be inherited from the father who had normal phenotype. If the genomic alternation is detected both in the affected parent and in the healthy one, it is clinically insignificant.^[18]^ Therefore, the pathogenicity of the region still remains unclear even after parental studies, because of incomplete penetrance or variable expressivity.

## 4 Conclusions

Since the URSMS is a rare congenital abnormality involving various organ systems, it is extremely difficult to be diagnosed prenatally. However, we can detect the following common manifestations on the antenatal sonography as suspicious signs of URSMS. According to our statistics from literatures, the signs include: severe oligohydramnios or anhydramnios, urinary tract anomalies, fetal intra-abdominal cysts, and dilated bowel, which are listed in order of ascending frequency. Additionally, it is worth noting that the enterolithiasis and vesicocolic fistulae are relatively infrequent, but highly specific features of URSMS. Consequently, a precise prenatal sonographic examination is crucial for diagnosing URSMS, and at the same time, more genomic profiling studies are needed as the causality of it is still unclear yet.

## Supplementary Material

Supplemental Digital Content
